# Supplementary material for: Therapeutic Properties and Use of Extra Virgin Olive Oil in Clinical Nutrition: A Narrative Review and Literature Update
Source: Nutrients. 2022 Mar 31;14(7):1440. doi: 10.3390/nu14071440 (PMC9003415; doi:10.3390/nu14071440)
Supplement: Supplementary file 1 [file nutrients-14-01440-s001.zip › nutrients-1622512-supplementary.pdf]

Supplementary Table S1. Composition of fatty acids in EVOO produced from different ~~Spanish~~ Mediterranean cultivars (expressed in percentages). Modified from [1–13].

Cultivar	Location	C14:0	C16:0	C16:1	C17:0	C17:1n-8	C18:0	C18:1n-9	C18:2n-6	C18:3n-3	C20:0	C20:1n-9	C22:0	C24:0	ΣMUFA	ΣPUFA
**Croatia**																
Lavtoska [2]	NR	NR	12.3 ± 1.0	0.9 ± 0.1	< LOD	0.1 ± 0.1	2.3 ± 0.0	72.1 ± 1.4	10.4 ± 0.3	0.7 ± 0.0	0.4 ± 0.0	0.3 ± 0.0	0.1 ± 0.0	NR	NR	NR
Leccino [1]	Šestanovac	NR	15.7 ± 0.0	1.4 ± 0.0	NR	0.1 ± 0.0	1.8 ± 0.0	72.1 ± 0.0	7.1 ± 0.0	0.9 ± 0.0	0.4 ± 0.0	0.3 ±	0.1 ± 0.0	0.1 ±	72.8 ± 0.1	10.5 ± 0.0
Leccino [1]	Kaštela	NR	16.1 ± 0.1	1.7 ± 0.1	NR	0.1 ± 0.0	1.9 ± 0.0	71.7 ± 0.0	6.7 ± 0.0	0.9 ± 0.0	0.5 ± 0.0	0.4 ± 0.0	0.1 ± 0.0	0.1 ±	69.2 ± 0.0	13.0 ± 0.0
Levantinka [2]	NR	NR	12.6 ± 1.3	0.9 ± 0.1	< LOD	0.1 ± 0.0	3.2 ± 0.4	73.0 ± 3.1	8.2 ± 1.1	0.8 ± 0.1	0.6 ± 0.1	0.3 ± 0.0	0.2 ± 0.0	NR	NR	NR
Oblica [2]	NR	NR	12.6 ±	0.9 ± 0.1	< LOD	0.1 ± 0.0	2.3 ± 0.2	70.2 ± 2.3	12.1 ± 1.0	0.7 ± 0.0	0.4 ± 0.0	0.3 ± 0.1	< LOD	NR	NR	NR
**Greece**																
Chondrolia, Chal-	Chalkidiki	NR	13.2	1.0	0.04	0.1	2.1	75.0	6.8	0.7	0.5	0.4	0.1	0.1	NR	NR
Koroneiki [2]	NR	NR	11.2 ± 0.6	0.9 ± 0.1	0.2 ±	0.1 ± 0.0	2.6 ± 0.2	77.7 ± 1.8	5.6 ± 1.1	0.5 ± 0.1	0.5 ± 0.1	0.5 ± 0.1	0.3 ± 0.1	NR	NR	NR
Koroneiki [4]	Crete	NR	11.9 ± 1.9	0.9 ± 0.0	0.0 ±	0.1 ± 0.0	2.8 ± 0.0	75.0 ± 1.1	6.1 ± 0.4	0.7 ± 0.0	0.4 ± 0.0	0.3 ± 0.0	0.0 ± 0.0	0.0 ±	NR	NR
Koroneiki (ripe) [5]	Argolida	0.0 ±	12.2 ± 0.2	1.3 ± 0.0	0.0 ±	0.1 ± 0.0	3.5 ± 0.1	70.6 ± 0.6	8.1 ± 0.1	0.8 ± 0.0	0.5 ± 0.0	0.0 ± 0.0	NR	0.1 ±	74.0 ± 0.6	9.4 ± 0.2
Koroneiki (green) [5]	Argolida	0.0 ±	10.8 ± 0.2	1.0 ± 0.0	00.0 ±	0.0 ± 0.0	4.6 ± 0.1	72.4 ± 0.6	7.2 ± 0.1	0.7 ± 0.0	0.4 ± 0.0	0.0 ± 0.0	NR	0.1 ±	75.6 ± 0.6	8.3 ± 0.2
Media Oblonga [2]	NR	NR	11.8 ± 0.1	0.6 ± 0.0	0.1 ±	0.2 ± 0.0	2.1 ± 0.0	62.0 ± 0.3	19.5 ± 1.1	0.7 ± 0.0	0.3 ± 0.0	0.3 ± 0.0	0.1 ± 0.0	NR	NR	NR
Megaritiki (ripe) [5]	Argolida	0.1 ±	14.3 ± 0.4	2.8 ± 0.1	0.0 ±	0.1 ± 0.0	2.5 ± 0.1	62.3 ± 0.7	11.6 ± 0.1	0.8 ± 0.0	0.4 ± 0.0	0.1 ± 0.0	0.1 ± 0.0	0.0 ±	69.2 ± 0.4	12.9 ± 0.3
Megaritiki (green) [5]	Argolida	0.0 ±	13.4 ± 0.3	2.2 ± 01	0.0 ±	0.1 ± 0.0	2.4 ± 0.1	63.9 ± 0.6	11.2 ± 0.1	0.8 ± 0.0	0.4 ± 0.0	0.2 ± 0.0	0.1 ± 0.0	0.2 ±	70.4 ± 0.5	12.5 ± 0.4
Throumbolia [4]	Crete	NR	12.3 ± 1.5	0.6 ± 0.0	0.1 ±	0.2 ± 0.1	2.0 ± 0.0	62.0 ± 1.0	19.5 ± 1.2	0.7 ± 0.1	0.3 ± 0.2	0.3 ± 0.2	0.1 ± 0.0	0.0 ±	NR	NR
**Italy**																
Castiglionese [2]	NR	NR	15.0	1.3	0.1	0.1	2.5	72.0	8.0	0.7	0.3	0.3	0.1	NR	NR	NR
Coratina [2]	NR	NR	9.1 ± 0.3	0.4 ± 0.1	0.1 ±	0.0 ± 0.0	2.4 ± 0.1	80.8 ± 1.0	6.1 ± 0.7	0.7 ± 0.0	0.4 ± 0.0	0.4 ± 0.0	0.1 ± 0.0	NR	NR	NR
Drita [2]	NR	NR	12.2	0.9	0.1	0.1	2.6	77.5	5.0	0.4	0.5	0.4	0.1	NR	NR	NR
Fantoio [2]	NR	NR	10.2 ± 1.2	0.6 ± 0.1	NR	0.1 ± 0.0	1.6 ± 0.2	80.6 ± 3.3	5.4 ± 1.1	0.6 ± 0.2	0.3 ± 0.1	0.4 ± 0.1	0.1 ± 0.0	NR	NR	NR
Leccino [2]	NR	NR	12.7 ± 1.1	1.2 ± 0.0	0.0 ±	0.1 ± 0.0	1.7 ± 0.1	76.0 ± 3.1	5.8 ± 1.5	0.6 ± 0.1	0.3 ± 0.0	0.2 ± 0.0	0.1 ± 0.0	NR	NR	NR
Maiatica [2]	NR	NR	11.2 ± 1.0	1.1 ± 0.1	0.0 ±	0.1 ± 0.0	3.1 ± 0.2	75.4 ± 1.6	7.5 ± 0.5	0.5 ± 0.0	0.4 ± 0.0	0.3 ± 0.0	0.1 ± 0.0	NR	NR	NR
Moraiolo [2]	NR	NR	11.0 ± 0.4	0.6 ± 0.1	0.0 ±	0.1 ± 0.0	1.8 ± 0.3	78.5 ± 1.7	6.1 ± 0.6	0.6 ± 0.1	0.3 ± 0.0	0.3 ± 0.1	0.1 ± 0.0	NR	NR	NR
Noccerala [2]	NR	NR	15.0	1.3	0.1	0.3	1.6	65.0	15.7	0.3	0.7	0.3	0.1	NR	NR	NR
Ogliarola [2]	NR	NR	18.2 ± 0.5	1.0 ± 0.2	0.0 ±	0.1 ± 0.0	2.0 ± 0.1	74.1 ± 1.1	8.6 ± 0.7	0.6 ± 0.0	0.4 ± 0.0	0.4 ± 0.0	0.1 ± 0.0	NR	NR	NR
Oliva Rossa [6]	Bari	0.0 ±	10.2 ± 0.0	0.5 ± 0.0	0.0 ±	0.1 ± 0.0	2.6 ± 0.0	76.2 ± 0.0	8.8 ± 0.0	0.8 ± 0.0	0.4 ± 0.0	0.4 ± 0.0	0.0 ± 0.0	0.0 ±	77.1 ± 0.0	9.6 ± 0.0
Oliva Rossa [6]	Bari	0.0 ±	10.3 ± 0.0	0.8 ± 0.0	0.1 ±	0.0 ± 0.0	2.6 ± 0.0	70.8 ± 0.2	13.5 ± 0.1	0.6 ± 0.0	0.6 ± 0.1	0.5 ± 0.0	0.1 ± 0.0	0.0 ±	72.1 ± 0.2	14.1 ± 0.1
Oliva Bianca [7]	Campania	NR	12.9 ± 0.2	0.9 ± 0.0	0.1 ±	NR	2.3 ± 0.0	74.8 ± 0.3	7.9 ± 0.1	0.7 ± 0.0	0.4 ± 0.0	NR	0.1 ± 0.0	NR	75.7 ± 0.3	8.6 ± 0.1
Ottobratica [2]	NR	NR	14.4	1.0	0.2	0.3	2.4	71.5	8.6	0.4	0.6	0.3	0.1	NR	NR	NR
Sinopolese [2]	NR	NR	13.2	0.9	0.1	0.2	2.4	76.1	5.2	0.4	0.8	0.3	0.2	NR	NR	NR
**Jordan**																
Nabali Baladi [2]	NR	NR	12.4 ± 0.1	1.1 ± 0.0	0.3 ±	0.3 ± 0.0	3.0 ± 0.2	67.1 ± 0.3	3.8 ± 0.2	0.7 ± 0.0	0.6 ± 0.0	0.3 ± 0.0	0.2 ± 0.0	NR	NR	NR
Nabali Muhasan [2]	NR	NR	12.5 ± 0.2	1.1 ± 0.0	0.3 ±	0.3 ± 0.0	3.1 ± 0.0	67.0 ± 0.4	13.9 ± 0.2	0.7 ± 0.0	0.6 ± 0.0	0.3 ± 0.0	0.2 ± 0.0	NR	NR	NR

Sahmi [2]	NR	NR	16.0 ± 0.3	1.2 ± 0.2	0.1 ±	0.2 ± 0.0	2.2 ± 0.1	67.1. ± 0.5	11.4 ± 0.3	0.7 ± 0.1	0.4 ± 0.0	0.3 ± 0.0	0.1 ± 0.0	NR	NR	NR

Supplementary Table S1. *Cont*.

Cultivar	Location	C14:0	C16:0	C16:1	C17:0	C17:1n-	C18:0	C18:1n-9	C18:2n-6	C18:3n-3	C20:0	C20:1n-	C22:0	C24:0	ΣMUFA	ΣPUFA
Lebanon																
Baladi [8]	Abdeh	NR	13.9	0.5	NR	NR	4.0	67.6	11.8	0.6	NR	NR	NR	NR	68.1	67.5
Baladi [8]	Abdeh	NR	14.0	0.5	NR	NR	4.2	67.1	12.0	0.6	NR	NR	NR	NR	12.4	12.7
Spain																
Arbequina [9]	Zaragoza	NR	14.5 ± 0.0	1.4 ± 0.0	0.1 ±	0.2 ± 0.0	2.0 ± 0.0	70.1 ± 0.0	10.3 ± 0.0	0.6 ± 0.0	0.4 ± 0.0	NR	0.1 ± 0.0	NR	71.8	11.1
Arbequina [10]	Extremadura	0.0 ±	15.3 ± 1.4	1.6 ± 0.4	0.1 ±	0.23 ±	1.33 ±	67.2 ± 4.3	12.7 ± 2.6	0.6 ± 0.1	0.3 ± 0.1	0.3 ± 0.1	NR	NR	69.5 ± 4.1	13.3 ± 2.7
Arbequina [2]	NR	NR	12.5 ± 3.8	1.4 ± 0.3	0.1 ±	0.2 ± 0.0	1.4 ± 0.4	72.1 ± 3.4	11.4 ± 0.2	0.7 ± 0.1	0.4 ± 0.1	0.35 ±	0.1 ± 0.0	NR	NR	NR
Carrasqueña [2]	NR	NR	11.4 ± 0.3	1.2 ± 0.1	0.0 ± 0	0.1 ± 0.0	2.8 ± 0.1	67.0 ± 1.1	16.0 ± 2.1	0.9 ± 0.0	0.4 ± 0.0	0.5 ± 0.0	0.3 ± 0.0	NR	NR	NR
Cornicabra [2]	NR	NR	8.6 ± 1.3	0.7 ± 0.1	0.1 ±	0.1 ± 0.0	2.5 ± 0.1	78.7 ± 5.1	7.5 ± 3.9	0.7 ± 0.1	0.5 ± 0.0	0.4 ± 0.0	0.1 ± 0.0	NR	NR	NR
Cornicabra [9]	Toledo	NR	8.9 ± 0.1	0.6 ± 0.0	0.1 ±	0.1 ± 0.0	3.2 ± 0.0	82.5 ± 0.1	3.3 ± 0.0	0.6 ± 0.0	0.5 ± 0.0	NR	0.2 ± 0.0	NR	83.2	4.1
Cornicabra [10]	Extremadura	0.0 ±	12.1 ± 2.1	1.0 ± 0.3	0.1 ±	0.1 ± 0.0	2.4 ± 1.1	77.4 ± 6.1	5.3 ± 4.2	0.7 ± 0.2	0.4 ± 0.1	0.3 ± 0.0	NR	NR	78.7 ± 6.1	6.0 ± 4.3
Cuquillo [9]	Murcia	NR	11.2 ± 0.0	0.8 ± 0.0	0.1 ±	0.1 ± 0.0	3.1 ± 0.0	75.4 ± 0.0	7.7 ± 0.0	0.8 ± 0.0	0.4 ± 0.0	NR	0.1 ± 0.0	NR	76.4	8.7
Empeltre [9]	Teruel	NR	11.1 ± 0.0	0.9 ± 0.0	0.1 ±	0.2 ± 0.0	1.7 ± 0.0	72.9 ± 0.0	11.6 ± 0.0	0.8 ± 0.0	0.3 ± 0.0	NR	0.1 ± 0.0	NR	74.0	12.7
Empeltre [2]	NR	NR	9.6 ± 0.4	0.8 ± 0.0	0.1 ± 0	0.2 ± 0.0	1.2 ± 0.0	77.5 ± 1.0	9.1 ± 0.6	0.6 ± 0.0	0.4 ± 0.0	0.4 ± 0.0	0.1 ± 0.0	NR	NR	NR
Hojiblanca [2]	NR	NR	9.3 ± 1.2	0.7 ± 0.2	0.1 ±	0.2 ± 0.1	2.5 ± 0.6	77.6 ± 3.0	8.2 ± 1.8	0.6 ± 0.2	0.3 ± 0.1	0.3 ± 0.1	0.1 ± 0.0	NR	NR	NR
Hojiblanca [9]	Jaén	NR	10.6 ± 0.1	0.9 ± 0.0	0.1 ±	0.1 ± 0.0	3.7 ± 0.0	77.5 ± 0.1	5.7 ± 0.0	0.7 ± 0.0	0.4 ± 0.0	NR	0.1 ± 0.0	NR	78.5	6.6
Lechín [2]	Sevilla	NR	11.0 ± 1.1	1.1 ± 0.1	0.1 ±	0.2 ± 0.0	2.1 ± 1.0	74.5 ± 1.4	9.4 ± 1.1	0.7 ± 0.2	0.3 ± 0.1	0.3 ± 0.1	0.1 ± 0.1	NR	NR	NR
Lechin [9]	Sevilla	NR	12.3 ± 0.0	1.0 ± 0.0	0.1 ±	0.2 ± 0.0	3.0 ± 0.0	73.8 ± 0.0	8.0 ± 0.0	0.9 ± 0.0	0.4 ± 0.0	NR	0.0 ± 0.0	NR	75.0	9.1
Manzanilla [2]	NR	NR	10.6 ± 1.3	1.0 ± 0.3	0.1 ±	0.2 ± 0.1	2.5 ± 0.2	75.6 ± 3.3	8.1 ± 1.6	0.7 ± 0.2	0.5 ± 0.2	0.4 ± 0.1	0.1 ± 0.0	NR	NR	NR
Manzanilla [2]	NR	NR	12.5 ± 0.6	1.1 ± 0.4	0.1 ±	0.1 ± 0.0	1.7 ± 0.2	68.3 ± 0.9	14.1 ± 0.9	1.1 ± 0.4	0.4 ± 0.1	0.3 ± 0.1	0.1 ± 0.0	NR	NR	NR
Manzanilla [9]	Cáceres	NR	10.1 ± 0.1	0.9 ± 0.0	0.1 ±	0.1 ± 0.0	2.8 ± 0.0	79.2 ± 0.0	5.5 ± 0.0	0.8 ± 0.0	0.5 ± 0.0	NR	0.0 ± 0.0	NR	80.1	6.5
Manzanilla [10]	Extremadura	0.0 ±	12.3 ± 1.2	1.12 ±	0.0 ±	0.1 ± 0.0	1.7 ± 0.7	78.4 ± 3.4	4.8 ± 2.4	0.7 ± 0.1	0.4 ± 0.0	0.3 ± 0.0	NR	NR	79.9 ± 3.3	5.5 ± 2.4
Manzanilla [10]	Extremadura	0.0 ±	13.2 ± 1.4	1.3 ± 0.2	0.2 ±	0.3 ± 0.1	2.8 ± 1.0	74.6 ± 2.7	6.0 ± 2.1	0.6 ± 0.2	0.5 ± 0.0	0.3 ± 0.1	NR	NR	76.5 ± 2.7	6.6 ± 2.2
Morisca [10]	Extremadura	0.0 ±	13.9 ± 0.9	1.1 ± 0.2	0.1 ±	0.1 ± 0.0	2.7 ± 0.8	65.9 ± 3.2	14.5 ± 2.4	0.9 ± 0.1	0.4 ± 0.0	0.2 ± 0.0	NR	NR	67.3 ± 3.1	15.4 ± 2.4
Pico Limón [2]	NR	NR	9.1 ± 0.2	0.9 ± 0.1	0.2 ±	0.2 ± 0.0	2.4 ± 0.1	78.1 ± 2.1	8.0 ± 1.1	0.7 ± 0.0	0.5 ± 0.0	0.3 ± 0.0	0.1 ± 0.0	NR	NR	NR
Pico Limón [10]	Extremadura	0.0 ±	13.2 ± 1.3	1.2 ± 0.2	0.0 ±	0.1 ± 0.1	2.1 ± 0.6	74.1 ± 3.2	7.7 ± 2.4	0.6 ± 0.1	0.4 ± 0.0	0.2 ± 0.0	NR	NR	75.7 ± 3.1	8.3 ± 2.5
Picual [2]	NR	NR	9.6 ± 1.0	0.8 ± 0.2	0.0 ±	0.1 ± 0.0	2.8 ± 0.6	80.8 ± 3.1	4.6 ± 1.3	0.5 ± 0.1	0.3 ± 0.1	0.2 ± 0.1	0.0 ± 0.0	NR	NR	NR
Picual [10]	Extremadura	0.0 ±	11.6 ± 1.2	1.0 ± 0.2	0.0 ±	0.1 ± 0.0	2.0 ± 0.9	80.7 ± 1.9	3.1 ± 0.5	0.6 ± 0.1	0.4 ± 0.0	0.3 ± 0.0	NR	NR	82.1 ± 1.9	3.7 ± 0.5
Picual [9]	Jaén	NR	10.1 ± 0.0	0.8 ± 0.0	0.1 ±	0.1 ± 0.0	3.7 ± 0.0	78.7 ± 0.0	5.1 ± 0.0	0.9 ± 0.0	0.5 ± 0.0	NR	0.1 ± 0.0	NR	79.6	6.0
Picudo [2]	NR	NR	10.3 ± 0.7	0.9 ± 0.2	0.1 ±	0.1 ± 0.0	2.7 ± 0.4	76.6 ± 3.2	8.0 ± 2.2	0.5 ± 0.2	0.4 ± 0.1	0.3 ± 0.1	0.1 ± 0.0	NR	NR	NR
Picudo [9]	Córdoba	NR	12.8 ± 0.0	1.2 ± 0.0	0.1 ±	0.1 ± 0.0	2.1 ± 0.0	72.8 ± 0.1	9.3 ± 0.0	1.0 ± 0.0	0.4 ± 0.0	NR	0.1 ± 0.0	NR	74.2	10.4
Verdial [10]	Extremadura	0.0 ±	13.5 ± 1.7	0.8 ± 0.3	0.1 ±	0.1 ± 0.0	2.9 ± 1.0	63.2 ± 3.6	17.5 ± 3.3	0.8 ± 0.2	0.5 ± 0.0	0.4 ± 0.1	NR	NR	64.4 ± 3.4	18.3 ± 3.3
Verdial [2]	Sevilla	NR	10.2 ± 0.5	0.7 ± 0.1	0.2 ±	0.3 ± 0.0	2.5 ± 0.2	74.7 ± 1.8	9.0 ± 1.1	0.8 ± 0.1	0.5 ± 0.0	0.5 ± 0.1	0.2 ± 0.0	NR	NR	NR

Verdial [2]	Málaga	NR	9.6 ± 0.2	0.6 ± 0.1	0.1 ±	0.2 ± 0.0	2.1 ± 0.2	73.4 ± 1.9	10.9 ± 1.2	1.2 ± 0.2	0.5 ± 0.0	0.4 ± 0.1	0.1 ± 0.0	NR	NR	NR

Supplementary Table S1. *Cont*.

Cultivar	Location	C14:0	C16:0	C16:1	C17:0	C17:1n-	C18:0	C18:1n-9	C18:2n-6	C18:3n-3	C20:0	C20:1n-	C22:0	C24:0	ΣMUFA	ΣPUFA
Tunisia																
Aloui [11]	"North"	NR	11.0 ± 2.2	0.6 ± 0.4	NR	NR	3.0 ± 1.0	67.2 ± 5.4	17.2 ± 3.9	0.7 ± 0.0	0.4 ± 0.0	NR	NR	NR	NR	NR
Baldi [13]	Gafsa	NR	16.5 ± 0.0	0.1 ± 0.0	0.1±0.0	0.2±0.0	2.9	63.6 ± 0.1	13.5 ± 0.0	0.9 ± 0.1	0.5 ± 0.0	0.3	0.1	0.1 ±	65.3 ± 0.1	14.4 ± 0.0
Besbessi [13]	Gafsa	NR	16.4 ± 0.3	0.1	0.1	0.2±0.0	1.4 ± 0.1	64.7 ± 0.8	14.8 ± 0.4	0.8 ± 0.0	0.3 ± 0.0	0.2 ± 0.0	0.1	0.1 ±	66.1 ± 0.7	15.6 ± 0.4
Chemchali [13]	Sidi Bouzid	NR	14.2 ± 0.4	0.1 ± 0.0	0.1 ±	0.1 ± 0.0	1.8 ± 0.1	67.0 ±	14.6 ± 0.4	0.7 ± 0.0	0.4 ± 0.0	0.3 ± 0.0	0.1 ± 0.0	0.1 ±	68.1 ± 0.4	15.34 ± 0.4
Chemchali [13]	Gafsa	NR	18.1 ± 0.4	0.0	0.0	0.1	2.1 ± 0.1	62.1 ± 0.3	14.0 ± 0.1	0.7 ± 0.0	0.4 ± 0.0	0.3 ± 0.0	0.1 ± 0.0	0.1	64.3 ± 0.4	14.7 ± 0.1
Chemchali [13]	Gafsa	NR	14.8 ± 0.8	0.0	0.0	0.0	2.0 ± 0.0	67.5 ± 0.6	13.0 ± 0.2	0.6 ± 0.0	0.4 ± 0.0	0.2 ± 0.0	0.1 ± 0.0	0.1	69.0 ± 0.5	13.6 ± 0.2
Chemlali [11]	"Center"	NR	18.6 ± 0.3	2.2 ± 0.2	NR	NR	2.7 ± 0.9	55.9 ± 0.6	18.0 ± 0.5	1.0 ± 0.1	0.5 ± 0.0	NR	NR	NR	58.1 ± 0.6	19.0 ± 0.8
Chemlali [12]	Hammamet	NR	15.5 ± 0.1	1.6 ± 0.1	0.1 ±	0.2 ± 0.0	2.0 ± 0.1	65.5 ± 0.5	13.5 ± 1.5	0.4 ± 0.0	0.3 ± 0.0	0.7 ± 0.0	NR	NR	68.1 ± 0.6	14.0 ± 1.5
Chemlali [12]	Gafsa	NR	14.8 ± 0.5	1.8 ± 0.1	0.0 ±	0.1 ± 0.0	2.2 ± 0.2	66.2 ± 0.4	13.5 ± 1.0	0.4 ± 0.0	0.3 ± 0.0	0.6 ± 0.0	NR	NR	68.7 ± 0.5	13.9 ± 1.1
Chemlali [12]	Kairouan	NR	16.9 ± 0.8	2.2 ± 0.1	0.0 ±	0.1 ± 0.0	2.4 ± 0.0	60.2 ± 0.2	16.9 ± 0.7	0.4 ± 0.0	0.2 ± 0.0	0.7 ± 0.0	NR	NR	63.1 ± 0.4	17.3 ± 0.7
Chemlali [12]	Sfax	NR	19.5 ± 0.5	2.7 ± 0.0	0.0 ±	0.1 ± 0.0	2.7 ± 0.0	56.1 ± 1.0	17.5 ± 1.2	0.5 ± 0.0	0.2 ± 0.0	0.7 ± 0.0	NR	NR	59.6 ± 1.0	17.9 ± 1.2
Chemlali [12]	Zarzis	NR	18.7 ± 0.0	2.3 ± 0.1	0.0 ±	0.1 ± 0.0	2.9 ± 0.0	61.8 ± 0.3	12.9 ± 0.3	0.4 ± 0.0	0.2± 0.0	0.7 ± 0.0	NR	NR	64.9 ± 0.4	13.3 ± 0.3
Chetoui [11]	"Center"	NR	13.5 ± 0.2	0.3 ± 0.0	NR	NR	2.3 ± 0.2	68.8 ± 0.4	14.0 ± 0.3	0.5 ± 0.0	0.4 ± 0.0	NR	NR	NR	69.2 ± 0.2	15.6 ± 0.2
Chetoui [11]	"Center"	NR	18.6 ± 0.3	2.2 ± 0.2	NR	NR	2.7 ± 0.9	55.9 ± 0.6	18.0 ± 0.5	1.0 ± 0.1	0.5 ± 0.0	NR	NR	NR	58.1 ± 0.6	19.0 ± 0.8
Chladmi [11]	"North"	NR	14.9 ± 2.1	1.7 ± 0.5	NR	NR	2.8 ± 0.1	69.8 ± 0.9	9.8 ± 3.6	0.6 ± 0.1	0.4 ± 0.0	NR	NR	NR	NR	NR
Harboui [11]	"North"	NR	14.5 ± 1.8	0.7 ± 0.2	0.0 ±	0.0 ± 0.0	2.1 ± 0.3	62.8 ± 3.3	18.4 ± 1.3	0.8 ± 0.1	0.4 ± 0.0	0.2 ± 0.0	0.1 ± 0.0	NR	NR	NR
Neb Jadmel [11]	"North"	NR	16.5	1.3	0.0	0.0	2.7	71.0	10.9	0.7	0.5	0.2	0.1	NR	NR	NR
Neb Jmel [13]	Gafsa	NR	17.4 ± 0.0	0.1	0.1	0.1±0.0	2.1 ± 0.0	65.8 ± 0.0	11.3 ± 0.0	0.9 ± 0.0	0.5	0.3 ± 0.0	0.1	0.1	67.6 ± 0.0	12.1 ± 0.0
Regregui [11]	"North"	NR	15.4 ± 1.0	0.8 ± 0.2	0.0 ±	0.0 ± 0.0	1.9 ± 0.1	62.4 ± 1.3	18.3 ± 0.5	0.7 ± 0.2	0.4 ± 0.0	0.2 ± 0.0	0.1 ± 0.0	NR	NR	NR
Rekahmi [11]	"North"	NR	14.9 ± 2.2	0.6 ± 0.3	0.0 ±	0.0 ± 0.0	2.4 ± 0.6	64.6 ± 5.6	16.1 ± 5.1	0.8 ± 0.1	0.4 ± 0.1	0.2 ± 0.1	0.1 ± 0.0	NR	NR	NR
Sayali [11]	"North"	NR	11.0	0.2	NR	NR	2.7	77.4	5.9	1.7	0.2	0.6	NR	NR	NR	NR
Sehli [13]	Sidi Bouzid	NR	14.0 ± 0.2	0.0 ± 0.0	0.0 ±	0.0	2.1 ± 0.0	69.7 ± 0.4	11.4 ± 0.2	0.7 ± 0.0	0.4 ± 0.0	0.2 ± 0.0	0.1	0.06	71.3 ± 0.4	12.0 ± 0.2
Sehli [13]	Sidi Bouzid	NR	13.5 ± 0.4	0.0	0.0	0.0	1.9 ± 0.0	71.9 ± 0.8	10.1 ± 0.3	0.5 ± 0.0	0.4 ± 0.0	0.2 ± 0.0	0.1 ± 0.0	0.1	73.4 ± 0.8	10.7 ± 0.3
Sredki [11]	"North"	NR	10.5 ± 0.7	0.5 ± 0.0	NR	NR	2.4 ± 0.2	74.0 ± 0.8	11.6 ± 1.3	0.6 ± 0.0	0.4 ± 0.1	NR	NR	NR	NR	NR
Tounssi [13]	Gafsa	NR	16.4 ± 0.0	0.1	0.1	0.1±0.0	2.9 ± 0.0	67.7 ± 0.0	9.5 ± 0.1	0.9 ± 0.0	0.6	0.3 ± 0.0	0.2 ± 0.0	0.1	69.3 ± 0.0	10.4 ± 0.0

LOD: Limit of detection; NR: Not reported

1. Jukić Špika, M.; Perica, S.; Žanetić, M.; Škevin, D. Virgin olive oil phenols, fatty acid composition and sensory profile: can cultivar overpower environmental and ripening effect? *Antioxidants* **2021**, *10*, 689, doi:10.3390/antiox10050689.
2. Aparicio, R.; Harwood, J.L. *Handbook of Olive Oil: Analysis and Properties*, 2nd ed.; Springer: New York, NY, USA, 2013; ISBN 978-1-4614-7776-1.

3. Mastralexi, A.; Tsimidou, M.Z. The potential of virgin olive oil from cv. chondrolia chalkidikis and chalkidiki (greece) to bear he alth claims according to the European legislation. *Molecules* **2021**, *26*, 3184, doi:10.3390/molecules26113184.
4. Vekiari, S.A.; Oreopoulou, V.; Kourkoutas, Y.; Kamoun, N.; Msallem, M.; Psimouli, V.; Arapoglou, D. Characterization and seasonal variation of the quality of virgin olive oil of the throumbolia and koroneiki varieties from Southern Greece. *Grasas y Aceites* **2010**, *61*, 221–231, doi:10.3989/gya.108709.
5. Katsoyannos, E.; Batrinou, A.; Chatzilazarou, A.; Bratakos, S.M.; Stamatopoulos, K.; Sinanoglou, V.J. Quality parameters of olive oil from stoned and nonstoned koroneiki and megaritiki greek olive varieties at different maturity levels. *Grasas y Aceites* **2015**, *66*, e067–e067, doi:10.3989/gya.0711142.
6. Squeo, G.; Silletti, R.; Mangini, G.; Summo, C.; Caponio, F. The potential of apulian olive biodiversity: the case of oliva rossa virgin olive oil. *Foods* **2021**, *10*, 369, doi:10.3390/foods10020369.
7. Di Vaio, C.; Graziani, G.; Gaspari, A.; De Luca, L.; Aiello, A.; Cirillo, A.; Bruno, A.; Romano, R.; Ritieni, A. Drupe characters, fatty acids, polyphenolic and aromatic profile of olive oil obtained from "oliva bianca", minor autochthonous cultivar of Campania. *Plants* **2021**, *10*, 1119, doi:10.3390/plants10061119.
8. El Riachy, M.; Hamade, A.; Ayoub, R.; Dandachi, F.; Chalak, L. Oil content, fatty acid and phenolic profiles of some olive varieties growing in Lebanon. *Front. Nutr.* **2019**, *6*, 94, doi:10.3389/fnut.2019.00094.
9. Ramos-Escudero, F.; Morales, M.T.; Asuero, A.G. Characterization of bioactive compounds from monovarietal virgin olive oils: relationship between phenolic compounds-antioxidant capacities. *Int. J. Food Prop.* **2015**, *18*, 348–358, doi:10.1080/10942912.2013.809542.
10. Montaño, A.; Hernández, M.; Garrido, I.; Llerena, J.; Espinosa, F. Fatty acid and phenolic compound concentrations in eight different monovarietal virgin olive oils from Extremadura and the relationship with oxidative stability. *IJMS* **2016**, *17*, 1960, doi:10.3390/ijms17111960.
11. Youssef, O.; Guido, F.; Daoud, D.; Mokhtar, Z. Effect of cultivar on minor components in Tunisia olive fruits cultivated in microclimate. *Afr. J. Wood Sci. For.* **2020**, *8*, 001–008.
12. Gargouri, B.; Ammar, S.; Zribi, A.; Mansour, A.B.; Bouaziz, M. Effect of growing region on quality characteristics and phenolic compounds of chemlali extra-virgin olive oils. *Acta Physiol Plant* **2013**, *35*, 2801–2812, doi:10.1007/s11738-013-1312-z.
13. Chtourou, F.; Valli, E.; Ben Mansour, A.; Bendini, A.; Gallina Toschi, T.; Bouaziz, M. Characterization of virgin olive oils obtained from minor Tunisian varieties for their valorization. *Food Measure* **2021**, *15*, 5060–5070, doi:10.1007/s11694-021-01066-7.

Supplementary Table S2. Composition of antioxidants in EVOO from different Mediterranean cultivars (expressed in mg/kg of EVOO as mean ± sd if n > 1) . Modified from [1–24]

Cultivar	Location	HT	TY	VA	PCA	CA	OLE	OLEAG	OLEAC	LA	OLEOC	PINO	LU	TPC	α-T
							Algeria								
Mekki [1]	Birtouta	1.4 ± 0.0	9.0 ± 0.0	0.3 ± 0.0	0.0 ± 0.0	0.0 ± 0.0	0.0 ± 0.0	NR	NR	NR	NR	NR	NR	122.3 ± 0.0	NR
Aberkane [1]	Birtouta	1.2 ± 0.0	18.4 ± 0.0	0.4 ± 0.0	0.0 ± 0.0	0.0 ± 0.0	0.0 ± 0.0	NR	NR	NR	NR	NR	NR	137.2 ± 0.2	NR
Aghenaou [1]	Birtouta	2.4 ± 0.0	18.1 ± 0.0	0.4 ± 0.0	0.0 ± 0.0	0.0 ± 0.0	0.0 ± 0.0	NR	NR	NR	NR	NR	NR	191.5 ± 0.7	NR
Aghenfas [1]	Birtouta	3.9 ± 0.0	25.1 ± 0.0	0.3 ± 0.0	0.0 ± 0.0	0.0 ± 0.0	0.8 ± 0.0	NR	NR	NR	NR	NR	NR	228.7 ± 0.0	NR
Aimeli [1]	Birtouta	6.3 ± 0.0	18.3 ± 0.0	0.3 ± 0.0	0.0 ± 0.0	0.0 ± 0.0	0.0 ± 0.0	NR	NR	NR	NR	NR	NR	185.5 ± 0.1	NR
Blanquette [1]	Birtouta	9.0 ± 0.0	21.5 ± 0.1	0.6 ± 0.0	0.0 ± 0.0	0.0 ± 0.0	1.3 ± 0.1	NR	NR	NR	NR	NR	NR	218.9 ± 0.0	NR
Bouchouk [1]	Birtouta	4.2 ± 0.0	14.5 ± 0.0	0.3 ± 0.0	0.0 ± 0.0	0.0 ± 0.0	0.3 ± 0.0	NR	NR	NR	NR	NR	NR	199.4 ± 0.0	NR
Boughenfas [1]	Birtouta	2.8 ± 0.0	26.3 ± 0.0	0.2 ± 0.0	0.0 ± 0.0	0.0 ± 0.0	1.6 ± 0.0	NR	NR	NR	NR	NR	NR	167.6 ± 0.0	NR
Bouichret [1]	Birtouta	4.2 ± 0.0	13.5 ± 0.0	0.2 ± 0.0	0.1 ± 0.0	0.1 ± 0.0	0.0 ± 0.0	NR	NR	NR	NR	NR	NR	194.1 ± 0.1	NR
Bounguergueb [1]	Birtouta	3.5 ± 0.0	14.1 ± 0.0	1.0 ± 0.0	0.0 ± 0.0	0.7 ± 0.0	0.0 ± 0.0	NR	NR	NR	NR	NR	NR	145.4 ± 0.0	NR
Chemlal [1]	Birtouta	4.2 ± 0..0	19.2 ± 0.0	0.6 ± 0.0	0.1 ± 0.0	0.0 ± 0.0	0.0 ± 0.0	NR	NR	NR	NR	NR	NR	235.8 ± 0.0	NR
Grosse du Hama [1]	Birtouta	14.9 ± 0.0	33.0 ± 0.0	0.2 ± 0.0	0.0 ± 0.0	0.0 ± 0.0	0.4 ± 0.0	NR	NR	NR	NR	NR	NR	322.2 ± 0.0	NR
Hamra [1]	Birtouta	4.1 ± 0.0	20.7 ± 0.0	1.7 ± 0..0	0.2 ± 0.0	0.2 ± 0.0	13.1 ± 0.0	NR	NR	NR	NR	NR	NR	166.0 ± 0.0	NR
Limli [1]	Birtouta	2.9 ± 0.0	18.2 ± 0.0	0.2 ± 0.0	0.0 ± 0.0	0.0 ± 0.0	0.0 ± 0.0	NR	NR	NR	NR	NR	NR	128.5 ± 0.1	NR
Neb djemel [1]	Birtouta	4.4 ± 0.0	17.1 ± 0. 0	0.3 ± 0.0	0.0 ± 0.0	0.0 ± 0.0	0.0 ± 0.0	NR	NR	NR	NR	NR	NR	194.0 ± 0.0	NR
Ronde de miliana [1]	Birtouta	0.0 ± 0.0	20.1 ± 0.0	0.7 ± 0.0	0.0 ± 0.0	0.1 ± 0.0	1.5 ± 0.0	NR	NR	NR	NR	NR	NR	235.1 ± 0.0	NR
Rougette de la mitidja [1]	Birtouta	4.6 ± 0.0	19.6 ± 0.0	0.2 ± 0.0	0.3 ± 0.0	0.1 ± 0.0	1.2 ± 0.0	NR	NR	NR	NR	NR	NR	113.4 ± 0.1	NR
Sigoise [1]	Birtouta	1.4 ± 0.0	27.8 ± 0.0	0.1 ± 0.0	0.0 ± 0.0	0.0 ± 0.0	0.2 ± 0.0	NR	NR	NR	NR	NR	NR	234.2 ± 0.0	NR
							Croatia								
Oblica [2]	Ugljan	18.2 ± 3.0	8.1 ± 0.3	0.6 ± 0.2	0.4 ± 0.0	0.8 ± 0.2	NR	NR	300.0 ± 32.0	NR	NR	4.0 ± 0.1	NR	506.0 ± 63.0	NR
							Greece								
Athenolia [3]	Molaoi	NR	NR	NR	NR	NR	NR	42.0 ± 0.0	80.0 ± 10.0	41.0 ± 3.0	99.0 ± 4.0	NR	NR	1320.0 ± 60.0	NR
Kalamata [3]	Molaoi	NR	NR	NR	NR	NR	NR	5.0 ± 0.0	202.0 ± 9.0	5.0 ± 0.0	748.0 ± 52.0	NR	NR	1000.0 ± 67.0	NR
Koroneiki [3]	Molaoi	NR	NR	NR	NR	NR	NR	NR	135.0 ± 3.0	NR	150.0 ± 11.0	NR	NR	878.0 ± 60.0	NR

Supplementary Table S2. *Cont*

Cultivar	Location	Location	HT	TY	VA	PCA	CA	OLE	OLEAG	OLEAC	LA	OLEOC	PINO	LU	TPC
Koroneiki [4]	Rethymnon	10.9 ± 0.2	4.9 ± 0.1	NR	NR	NR	NR	7.0 ± 0.3	3.5 ± 0.2	NR	5.9 ± 0.0	NR	NR	NR	NR
Throubolia [3]	Molaoi	NR	NR	NR	NR	NR	NR	41.0 ± 3.0	22.0 ± 1.0	35.0 ± 3.0	20.0 ± 0.0	NR	NR	1550.0 ± 36.0	NR
Throumbolia [4]	Rethymnon	13.5 ± 0.9	4.5 ± 0.5	NR	NR	NR	NR	1.4 ± 0.0	1.2 ± 0.0	NR	8.9 ± 0.0	NR	NR	NR	NR
							Italy								
Barone di Monteprofico [5]	Apulia	9.5 ± 0.8	NR	NR	NR	NR	NR	4.3 ± 0.5	7.9 ± 0.1	4.3 ± 0.2	4.3 ± 0.2	18.6 ± 0.1	9.9 ± 0.7	202 ± 14	NR
Bosana [6]	Sardinia	55.0	30.3	NR	NR	NR	NR	778.0	213.1	165.4	6.2	NR	20.8	NR	130.5
Casaliva [6]	Garda	70.9	40.2	NR	NR	NR	NR	799.6	239.1	107.7	7.3	NR	30.6	NR	112.6
Cellina di Nardò [5]	Apulia	6.9 ± 0.9	NR	NR	NR	NR	NR	15.3 ± 0.2	73.6 ± 0.5	68.3 ± 0.5	68.3 ± 0.5	1.4 ± 0.4	8.0 ± 0.7	253 ± 7	NR
Colozzese [5]	Apulia	25.8 ± 0.6	NR	NR	NR	NR	NR	28.2 ± 0.7	39.0 ± 0.7	75.4 ± 0.2	75.4 ± 0.2	6.5 ± 0.4	7.6 ± 0.4	251 ± 12	NR
Cornola [5]	Apulia	1.8 ± 0.4	NR	NR	NR	NR	NR	33.1 ± 0.7	11.7 ± 0.2	10.2 ± 0.6	10.2 ± 0.6	1.6 ± 0.2	2.2 ± 0.5	189 ± 10	NR
Leccio del Corno [6]	Campania	140.0	43.5	NR	NR	NR	NR	896.8	279.2	193.0	16.2	NR	18.1	NR	176.2
Mixture [7]	Sicily	36.7 ± 20.7	23.1 ± 22.8	1.7 ± 0.8	1.7 ± 0.7	NR	347.6 ± 180.3	37.0 ± 17.0	374.8 ± 319.2	126.1 ± 43.3	NR	NR	12.3 ± 0.7	159.1 ± 45.2	NR
Mixture [7]	Puglia	21.8 ± 20.0	12.8 ± 17.3	1.9 ± 0.5	1.4 ± 0.1	NR	631.2 ± 216.3	187.2 ± 110.3	619.0 ± 171.1	434.7 ± 235.2	NR	NR	14.1 ± 1.3	335.2 ± 112.9	NR
Mixture [7]	Tuscany	13.5 ± 11.4	5.0 ± 4.4	1.6 ± 0.8	1.7 ± 0.7	NR	479.5 ± 151.1	294.7 ± 330.9	689.4 ± 385.3	327.0 ± 245.7	NR	NR	13.8 ± 0.9	348.2 ± 148.5	NR
Mixture [7]	Lazio	14.5 ± 8.0	6.0 ± 5.5	1.9 ± 0.8	1.7 ± 0.4	NR	565.0 ± 209.0	59.3 ± 41.8	840.2 ± 418.7	188.1 ± 25.0	NR	NR	14.2 ± 1.6	238.5 ± 70.1	NR
Ogliarola di Lecce [5]	Apulia	6.6 ± 0.8	NR	NR	NR	NR	NR	24.1 ± 0.4	64.9 ± 0.6	64.5 ± 0.8	64.5 ± 0.8	4.6 ± 0.6	7.6 ± 0.3 d	278 ± 9	NR
Oliva Bianca [8]	Campania	12.0 ± 0.6	16.4 ± 0.0	0.5 ± 0.0	1.1 ± 0.0	NR	<LOD	85.9 ± 0.5	33.4 ± 0.0	122.4 ± 3.9	255.2 ± 7.1	NR	17.2 ± 2.4	648.9 ± 2.8	NR
Oliva Grossa [5]	Apulia	12.1 ± 0.5	NR	NR	NR	NR	NR	34.3 ± 0.8	124.4 ± 0.5	103.4 ± 0.8	103.4 ± 0.8	21.9 ± 0.6	16.7 ± 0.6	273 ± 3	NR
Oliva Rossa [9]	Bari	0.4 ± 0.1	0.5 ± 0.0	0.2 ± 0.0	NR	NR	NR	NR	25.8 ± 4.6	8.6 ± 1.7	14.0 ± 0.8	9.9 ± 0.7	6.8 ± 0.6	NR	237.2 ± 4.5
Cultivar	Location	Location	HT	TY	VA	PCA	CA	OLE	OLEAG	OLEAC	LA	OLEOC	PINO	LU	TPC
Orniella [5]	Apulia	7.1 ± 0.1	NR	NR	NR	NR	NR	26.2 ± 0.8	13.2 ± 0.5	6.7 ± 0.7	6.7 ± 0.7	1.6 ± 0.4	13.2 ± 0.2	198 ± 3	NR
Quercetano [10]	Tuscany (hi-lls)	678.2 ± 24.3	771.8 ± 38.7	6.8 ± 0.3	3.2 ± 0.2	NR	NR	330.8 ± 9.4	803.3 ± 46.1	756.1 ± 54.3	1102.6 ± 71.3	1819.4 ± 52.0	64.3 ± 4.1	NR	NR
Quercetano [10]	Tuscany (plains)	2167.8 ± 93.3	1697.5 ± 123.0	4.0 ± 0.1	10.3 ± 0.7	NR	NR	244.5 ± 8.7	2610.9 ± 169.5	251.7 ± 20.0	1316.2 ± 95.0	664.6 ± 23.8	85.8 ± 6.2	NR	NR

Supplementary Table S2. *Cont*

Cultivar	Location	Location	HT	TY	VA	PCA	CA	OLE	OLEAG	OLEAC	LA	OLEOC	PINO	LU	TPC
Spina [5]	Apulia	0.9 ± 0.5	NR	NR	NR	NR	NR	52.6 ± 0.6	19.4 ± 0.3	12.4 ± 0.8	12.4 ± 0.8	3.4 ± 0.5	7.8 ± 0.5 d	138 ± 7	NR
							Lebanon								
Baladi [11]	Abdeh	4.3	3.2	2.8	2.2	NR	NR	NR	27.0	NR	73.5	NR	7.2	321	NR
Baladi [11]	Abdeh	3.7	2.2	2.7	1.9	NR	NR	NR	27.8	NR	48.4	NR	3.9	301	NR
							Morocco								
Arbequine [12]	Meknes	NR	22.0 ± 3.0	1.5 ± 0.3	NR	NR	3.9 ± 0.2	NR	NR	NR	NR	NR	4.1 ± 0.1	NR	NR
Arbosana, Arbequine) [12]	Meknes	NR	13.8 ± 0.0	4.5 ± 0.1	NR	NR	3.0 ± 0.5	NR	NR	NR	NR	NR	4.0 ± 0.3	NR	NR
Koroneiki [12]	Meknes	NR	14.0 ± 1.0	6.0 ± 2.0	NR	NR	5.8 ± 0.6	NR	NR	NR	NR	NR	1.0 ± 0.0	NR	NR
**Picholine** [13]	Taounate	6.4 ± 0.5	<LOD	NR	NR	NR	NR	231.3 ± 4.4	3.1 ± 0.1	57.3 ± 2.9	<LOD	NR	<LOQ	354.2	100.4 ± 0.1
**Picholine** [13]	Chefchaouen	40.0 ± 1.5	300.6 ± 11.3	NR	NR	NR	NR	198.3 ±_ 13.2	33.3 ± 1.9	21.4 ± 0.4	<LOQ	NR	6.4 ± 0.2	723.4	55.3 ± 0.3
**Picholine** [13]	Taza	2.8 ± 0.1	<LOD	NR	NR	NR	NR	18.6 ± 0.0	2.3 ± 0.0	1.2 ± 0.1	<LOQ	NR	7.8 ± 0.7	50.4	38. ± 0.1
Picholine [12]	Meknes	NR	29.4 ± 0.6	1.9 ± 0.3	NR	NR	5.6 ± 0.4	NR	NR	NR	NR	NR	2.1 ± 0.0	NR	NR
Picual [12]	Meknes	NR	12.0 ± 6.0	2.0 ± 1.0	NR	NR	1.5 ± 0.8	NR	NR	NR	NR	NR	2.0 ± 1.0	NR	NR
							Spain								
Arbequina [14]	Extremadura	1.6 ± 1.8	1.3 ± 1.0	1.2 ± 0.9	0.3 ± 0.3	NR	NR	25.0 ± 5.2	NR	NR	77.4 ± 84.3	NR	2.5 ± 5.5	200.2 ± 177.1	NR
Arbequina [15]	Zaragoza	2.5 ± 0.2	1.9 ± 0.7	0.7 ± 0.1	0.2 ± 0.1	15.5 ± 3.3	NR	11.6 ± 1.6	26.6 ± 4.8	2.8 ± 0.9	17.4 ± 2.1	6.2 ± 2.1	NR	367.6	187.9 ± 15.1
Arbequina [16]	Mallorca	42.0 ± 0.3	6.8 ± 0.2	0.2 ± 0.0	0.4 ± 0.1	0.0 ± 0.0	NR	5.5 ± 0.0	93.0 ± 4.9	16.3 ± 5.7	6.4 ± 3.9	2.5 ± 0.1	3.0 ± 0.2	180.9 ± 5.9	NR
Blanqueta [17]	Jaén	6.7 ± 0.0	3.6 ± 0.0	0.0 ± 0.0	0.1 ± 0.0	20.0 ± 0.1	NR	59.8 ± 0.3	1067 ± 6.0	37.2 ± 0.2	313.2 ± 1.8	2.0 ± 0.0	0.3 ± 0.0	NR	NR
Corbella [18]	Barcelona	0.7 ± 0.1	NR	NR	0.7 ± 0.0	NR	NR	135.9 ±3.0	NR	11.9 ± 0.5	NR	6.6 ± 0.7	0.3 ± 0.0	483.7 ± 26.6	NR
Cornicabra [14]	Extremadura	10.2 ± 12.7	9.3 ± 12.5	0.2 ± 0.3	0.3 ± 0.4	NR	NR	94.6 ± 107.9	NR	NR	230.4 ± 138.6	NR	1.0 ± 1.4	632.6 ± 405.9	NR
Cornicabra [15]	Toledo	8.1 ± 3.5	8.7 ± 0.2	1.5 ± 0.6	0.3 ± 0.0	3.3 ± 1.0	NR	13.4 ± 1.0	1.1 ± 0.0	6.1 ± 1.8	6.4 ± 0.8	6.3 ± 2.4	NR	214.9	241.4 ± 2.3
Cornicabra [16]	Toledo	34.5 ± 13.0	13.8 ± 3.1	0.2 ± 0.0	0.0 ± 0.0	0.0 ± 0.0	NR	25.4 ± 4.8	25.0 ± 4.2	21.2 ± 0.5	12.5 ± 11.9	1.9± 0.1	0.8 ± 0.0	138.9 ± 36.6	NR
Cuquillo [15]	Murcia	5.5 ± 0.5	5.4 ± 0.9	1.6 ± 0.2	0.2 ± 0.1	4.3 ± 1.8	NR	23.7 ± 4.1	11.1 ± 1.7	9.2 ± 2.4	9.4 ± 2.2	5.4 ± 0.3	NR	295.3	165.9 ± 9.6
Empeltre [15]	Teruel	4.3 ± 1.0	2.5 ± 0.3	1.2 ± 0.9	0.1 ± 0.0	11.4 ± 2.1	NR	4.8 ± 1.0	9.7 ± 0.6	5.4 ± 0.2	8.6 ± 1.8	4.3 ± 1.2	NR	195.2	344.3 ± 15.4
Habichuelero [17]	Jaén	3.7 ± 0.1	3.2 ± 0.1	0.0 ± 0.0	0.1 ± 0.0	5.4 ± 0.1	NR	84.4 ± 1.4	292.8 ± 4.9	107.0 ± 1.8	104.7 ± 1.8	0.6 ± 0.0	0.2 ± 0.0	NR	NR
Hojiblanca [15]	Jaén	0.8 ± 0.0	4.3 ± 0.9	2.7 ± 0.1	0.7 ± 0.1	2.9 ± 0.9	NR	5.6 ± 0.1	2.8 ± 0.9	7.3 ± 0.6	6.9 ± 0.2	4.8 ± 1.1	NR	210.9	286.8 ± 9.7
Hojiblanca [16]	Antequera	28.31	9.941	0.356	0.384	0.008	NR	10.18	56.14	3.9	4.747	0.738	1.561	127.7	NR
Hojiblanca [16]	Córdoba	28.7 ± 1.8	7.3 ± 2.8	0.5 ± 0.1	0.9 ± 0.1	0.0 ± 0.0	NR	21.0 ± 9.5	19.7 ± 5.7	23.3 ± 20.1	4.7 ± 2.0	0.3 ± 0.9	2.0 ± 1.2	115.2 ± 23.8	NR

Supplementary Table S2. *Cont*

Cultivar	Location	Location	HT	TY	VA	PCA	CA	OLE	OLEAG	OLEAC	LA	OLEOC	PINO	LU	TPC
Hojiblanca (organic) [19]	Córdoba	4.5 ± 1.1	NR	NR	0.7 ± 0.1	NR	NR	7.2 ± 1.0	NR	54.6 ± 10.6	186.7 ± 40.6	0.5 ± 0.1	22.7 ± 5.1	456.9 ± 56.7	NR
Hojiblanca (conventional) [19]	Córdoba	3.6 ± 1.3	NR	NR	1.0 ± 0.4	NR	NR	4.7 ± 0.4	NR	34.7 ± 8.0	132.1 ± 37.0	0.8 ± 0.1	19.3 ± 5.4	338.2 ± 43.0	NR
Lechin [15]	Sevilla	5.4 ± 1.8	4.1 ± 1.4	0.8 ± 0.1	0.1 ± 0.0	6.3 ± 2.3	NR	11.9 ± 3.6	7.2 ± 2.3	5.4 ± 0.9	8.7 ± 1.7	4.5 ± 1.0	NR	303.9	191.4 ± 8.1
Lechin [20]	Granada	3.9±0.0	7.3±0.1	0.5±0.0	0.1±0.0	NR	NR	0.3±0.0*	NR	NR	NR	3.5±0.0	NR	NR	264±17
Manzanilla [15]	Cáceres	1.9 ± 0.4	4.3 ± 1.3	1.2 ± 0.0	0.4 ± 0.1	1.3 ± 0.6		6.7 ± 2.0	11.2 ± 3.2	7.1 ± 1.6	14.9 ± 1.6	6.1 ± 0.9	NR	214.5	234.7 ± 14.9
Manzanilla [14]	Extremadura	8.5 ± 9.3	13.0 ± 8.5	0.5 ± 0.3	0.7 ± 0.4	NR	NR	89.2 ± 71.4	NR	NR	117.3 ± 123.0	NR	2.5 ± 3.7	468.8 ± 348.2	NR
Manzanilla [14]	Extremadura	6.2 ± 3.9	7.1 ± 2.8	1.2 ± 1.4	1.5 ± 0.7	NR	NR	150.7 ± 94.2	NR	NR	151.6 ± 93.4	NR	2.7 ± 4.0	497.5 ± 284.1	NR
Morisca [14]	Extremadura	6.7 ± 3.8	4.9 ± 2.0	0.5 ± 0.4	2.2 ± 1.3	NR	NR	47.4 ± 37.8	NR	NR	259.3 ± 184.1	NR	2.2 ± 2.8	550.2 ± 346.8	NR
Pico Limón [14]	Extremadura	12.4 ± 16.5	9.7 ± 13.7	0.7 ± 0.6	0.3 ± 0.5	NR	NR	77.7 ± 128.2	NR	NR	135.3 ± 123.3	NR	3.4 ± 5.4	406.8 ± 415.2	NR
Picual [14]	Extremadura	12.8 ± 9.6	7.8 ± 4.4	1.3 ± 0.7	0.7 ± 0.3	NR	NR	49.4 ± 61.0	NR	NR	95.4 ± 54.9	NR	4.7 ± 7.9	380.4 ± 252.8	NR
Picual [17]	Jaén	0.7 ± 0.0	2.2 ± 0.1	0.6 ± 0.0	0.3 ± 0.0	3.3 ± 0.1	NR	98.8 ± 2.9	174.0 ± 5.3	104.0 ± 3.1	31.6 ± 0.9	3.2 ± 0.0	4.7 ± 0.1	NR	NR
Picual [15]	Jaén	NR	4.0 ± 0.8	0.6 ± 0.2	0.2 ± 0.1	2.1 ± 0.2	NR	0.1 ± 0.0	0.1 ± 0.0	11.2 ± 0.9	5.8 ± 1.7	3.9 ± 0.9	NR	86.9	249.5 ± 22.1
Picudo [15]	Córdoba	5.9 ± 0.8	7.8 ± 3.8	1.4 ± 0.3	1.5 ± 0.0	4.6 ± 0.8	NR	10.2 ± 1.6	12.6 ± 2.9	9.5 ± 2.1	17.9 ± 1.6	7.8 ± 1.7	NR	339.9	194.2 ± 15.0
Picudo [16]	Córdoba	11.4 ± 1.9	24.0 ± 5.3	0.2 ± 0.0	0.4 ± 0.1	0.0 ± 0.0	NR	18.6 ± 2.7	33.8 ± 0.9	13.1 ± 9.8	6.4 ± 8.1	0.0 ± 0.0	1.6 ± 0.2	113.5 ± 19.5	NR
Picudo [16]	Granada	12.4	0.0	0.1	0.0	0.0	NR	43.0	27.6	6.8	3.5	0.2	1.9	101.8	NR
Sevillana [17]	Jaén	0.9 ± 0.0	2.44 ± 0.0	0.6 ± 0.0	0.3 ± 0.0	2.0 ± 0.0	NR	11.1 ± 0.0	104.4 ± 0.3	9.4 ± 0.0	25.1 ± 0.0	8.0 ± 0.0	2.9 ± 0.0	NR	NR
Verdial [14]	Extremadura	8.0 ± 10.8	10.0 ± 10.2	0.4 ± 0.4	1.0 ± 0.7	NR	NR	39.4 ± 39.8	NR	NR	187.7 ± 213.7	NR	3.5 ± 4.3	551.4 ± 497.1	NR
							Tunisia								
Arbequina [21]	Borj El Amri	2.7 ± 0.1	1.3 ± 0.0	0.6 ± 0.0	0.3 ± 0.0	NR	2.6 ± 0.0	NR	NR	0.2 ± 0.0	NR	4.9 ± 0.0	12.6 ± 0.2	109.2 ± 0.0	NR
Chemlali [22]	Hammamet	17.0 ± 0.1	40.5 ± 0.4	11.2 ± 0.2	1.5 ± 0.1	NR	21.7 ± 0.9	26.9 ± 0.8	8.2 ± 1.1	11.3 ± 1.1	6.6 ± 0.4	23.8 ± 1.3	4.9 ± 0.1	NR	NR
Chemlali [22]	Gafsa	9.6 ± 0.1	36.4 ± 0.3	1.8 ± 0.1	8.5 ± 1.2	NR	2.3 ± 0.1	26.3 ± 1.2	11.8 ± 0.6	3.9 ± 0.4	1.5 ± 0.1	2.7 ± 0.3	1.1 ± 0.1	NR	NR
Chemlali [22]	Kairouan	12.7 ± 0.1	25.1 ± 0.6	1.2 ± 0.9	1.4 ± 0.1	NR	12.3 ± 0.7	79.8 ± 1.4	23.9 ± 0.9	9.7 ± 0.9	2.0 ± 0.7	22.0 ± 0.6	5.8 ± 0.6	NR	NR
Chemlali [22]	Sfax	19.8 ± 0.2	16.0 ± 0.1	1.6 ± 0.4	1.5 ± 0.2	NR	7.5 ± 1.0	43.7 ± 0.7	3.3 ± 0.4	4.1 ± 0.4	1.1 ± 0.5	23.2 ± 0.3	5.9 ± 0.2	NR	NR
Chemlali [22]	Zarzis	24.1 ± 0.2	19.5 ± 0.2	1.4 ± 0.6	1.5 ± 0.4	NR	1.5 ± 0.4	60.2 ± 1.0	12.3 ± 0.8	6.1 ± 1.2	1.5 ± 0.1	2.8 ± 0.1	1.7 ± 0.1	NR	NR

Supplementary Table S2. *Cont*

Cultivar	Location	Location	HT	TY	VA	PCA	CA	OLE	OLEAG	OLEAC	LA	OLEOC	PINO	LU	TPC
Chetoui [21]	Borj El Amri	15.1 ± 0.2	23.2 ± 0.3	1.1 ± 0.0	0.8 ± 0.0	NR	2.2 ± 0.0	381.6 ± 15.5	NR	9.7 ± 0.4	NR	6.5 ± 0.1	13.2 ± 0.3	492.6 ± 15.0	NR
Neb Jmal [21]	Borj El Amri	6.2 ± 0.1	16.5 ± 0.1	2.1 ± 0.0	1.8 ± 0.0	NR	0.0 ± 0.0	30.6 ± 0.1	NR	12.2 ± 0.1	NR	0.0 ± 0.0	17.1 ± 0.2	298.5 ± 11.2	NR
Picholine [21]	Borj El Amri	22.6 ± 0.0	17.0 ± 0.1	0.4 ± 0.0	0.0 ± 0.0	NR	0.0 ± 0.0	NR	NR	17.3 ± 0.0	NR	0.0 ± 0.0	4.3 ± 0.0	418.7± 0.5	NR
Oueslati [23]	Jbel Rihan	5.8	2.3	NR	NR	NR	NR	537.8	2394.0	19.4	45.3	NR	6.5	NR	NR
Oueslati [23]	Ain Jloula	6.5	1.9	NR	NR	NR	NR	360.3	644.6	2.9	3.2	0.6	0.9	NR	NR
Oueslati [23]	Khit el Oued	5.1	1.8	NR	NR	NR	NR	330.1	1601.9	4.3	20.9	0.7	1.0	NR	NR
Oueslati [23]	Haffouz	6.1	2.1	NR	NR	NR	NR	461.4	1496.5	7.9	18.5	0.6	1.4	NR	NR
Oueslati [23]	Menzel Rais	5.9	3.1	NR	NR	NR	NR	270.2	910.0	14.0	11.5	0.7	1.4	NR	NR
Oueslati [23]	Ala	7.2	2.4	NR	NR	NR	NR	222.6	1834.1	9.9	16.1	1.2	0.7	NR	NR
Oueslati [23]	Sfax	3.8	2.3	NR	NR	NR	NR	385.2	828.2	5.2	7.9	0.7	0.9	NR	NR
Turkey															
Mixture (mainly Ay-valik) [24]	North Izmir	7.4 ± 6.7	4.9 ± 5.0	0.2± 0.1	0.3 ± 0.2	0.1 ± 0.0	NR	NR	NR	NR	NR	NR	1.1 ± 1.0	230.7 ± 55.3	NR
Mixture (mainly Me-mecik) [24]	South Izmir	3.9 ± 2.6	10.7 ± 7.4	0.1 ± 0.1	0.7 ± 0.5	0.7 ± 0.2	NR	NR	NR	NR	NR	NR	1.3 ± 0.9	287.3 ± 58.2	NR

* the determination includes both aldehyde forms of oleuropeinm; LOD: limit of detection; LOQ: limit of quantitation; HT: hydroxi-tyrosol (3,4-DHPEA); TY: tyrosol (p-HPEA); VA: vanillic acid; PCA: p-coumaric acid; CA: cinnamic acid; OLE: oleuropein (3,4-DHPEA-elenolic acid glucoside); OLEAG: oleuropein-aglycone (3,4-DHPEA-EA); OLEAC: oleacein (3,4-DHPEA-EDA); LA: ligstroside-aglycone (p-HPEA-EA); OLEOC: oleocanthal (p-HPEA-EDA); PINO: (+)-pinoresinol; LU: luteolin; TPC: total phenolic content; α-T: α-tocopherol

1. Douzane, M.; Tamendjari, A.; Abdi, A.K.; Daas, M.S.; Mehdid, F.; Bellal, M.M. Phenolic compounds in mono-cultivar extra virgin olive oils from algeria. *Grasas y Aceites* **2013**, *64*, 285–294, doi:10.3989/gya.072212.
2. Torić, J.; Barbarić, M.; Uršić, S.; Jakobušić Brala, C.; Karković Marković, A.; Zebić Avdičević, M.; Benčić, Đ. Antique traditional practice: phenolic profile of virgin olive oil obtained from fruits stored in seawater. *Foods* **2020**, *9*, 1347, doi:10.3390/foods9101347.
3. Diamantakos, P.; Giannara, T.; Skarkou, M.; Melliou, E.; Magiatis, P. Influence of harvest time and malaxation conditions on the concentration of individual phenols in extra virgin olive oil related to its healthy properties. *Molecules* **2020**, *25*, 2449, doi:10.3390/molecules25102449.
4. Vekiari, S.A.; Oreopoulou, V.; Kourkoutas, Y.; Kamoun, N.; Msallem, M.; Psimouli, V.; Arapoglou, D. Characterization and seasonal variation of the quality of virgin olive oil of the throumbolia and koroneiki varieties from Southern Greece. *Grasas y Aceites* **2010**, *61*, 221–231, doi:10.3989/gya.108709.
5. Negro, C.; Aprile, A.; Luvisi, A.; Nicolì, F.; Nutricati, E.; Vergine, M.; Miceli, A.; Blando, F.; Sabella, E.; De Bellis, L. Phenolic profile and antioxidant activity of Italian monovarietal extra virgin olive oils. *Antioxidants* **2019**, *8*, 161, doi:10.3390/antiox8060161.
6. Różańska, A.; Russo, M.; Cacciola, F.; Salafia, F.; Polkowska, Ż.; Dugo, P.; Mondello, L. Concentration of potentially bioactive compounds in Italian extra virgin olive oils from various sources by using LC-MS and multivariate data analysis. *Foods* **2020**, *9*, 1120, doi:10.3390/foods9081120.

7. Fanali, C.; Della Posta, S.; Vilmercati, A.; Dugo, L.; Russo, M.; Petitti, T.; Mondello, L.; de Gara, L. Extraction, analysis, and antioxidant activity evaluation of phenolic compounds in different italian extra-virgin olive oils. *Molecules* **2018**, *23*, 3249, doi:10.3390/molecules23123249. 40
8. Di Vaio, C.; Graziani, G.; Gaspari, A.; De Luca, L.; Aiello, A.; Cirillo, A.; Bruno, A.; Romano, R.; Ritieni, A. Drupe characters, fatty acids, polyphenolic and aromatic profile of olive oil obtained from "oliva bianca", minor autochthonous cultivar of Campania. *Plants* **2021**, *10*, 1119, doi:10.3390/plants10061119.
9. Squeo, G.; Silletti, R.; Mangini, G.; Summo, C.; Caponio, F. The potential of apulian olive biodiversity: the case of oliva rossa virgin olive oil. *Foods* **2021**, *10*, 369, doi:10.3390/foods10020369.
10. Barbalace, M.C.; Zallocco, L.; Beghelli, D.; Ronci, M.; Scortichini, S.; Digiacomo, M.; Macchia, M.; Mazzoni, M.R.; Fiorini, D.; Lucacchini, A.; et al. Antioxidant and neuroprotective activity of extra virgin olive oil extracts obtained from quercetano cultivar trees grown in different areas of the Tuscany region (Italy). *Antioxidants* **2021**, *10*, 421, doi:10.3390/antiox10030421.
11. El Riachy, M.; Hamade, A.; Ayoub, R.; Dandachi, F.; Chalak, L. Oil content, fatty acid and phenolic profiles of some olive varieties growing in Lebanon. *Front. Nutr.* **2019**, *6*, 94, doi:10.3389/fnut.2019.00094.
12. El Hilali, H.; El Hilali, F.; Porter, S.E.G.; Ghali, S.A.; Meyls, H.M.; Ouazzani, N.; Laziri, F.; Barber, A. Olive oil varieties cultivated in Morocco reduce reactive oxygen species and cell viability of human cervical cancer cells. *MNM* **2020**, *13*, 89–100, doi:10.3233/MNM-190390.
13. Bouymajane, A.; Oulad El Majdoub, Y.; Cacciola, F.; Russo, M.; Salafia, F.; Trozzi, A.; Rhazi Filali, F.; Dugo, P.; Mondello, L. Characterization of phenolic compounds, vitamin E and fatty acids from monovarietal virgin olive oils of "picholine marocaine" cultivar. *Molecules* **2020**, *25*, 5428, doi:10.3390/molecules25225428.
14. Montaño, A.; Hernández, M.; Garrido, I.; Llerena, J.; Espinosa, F. Fatty acid and phenolic compound concentrations in eight different monovarietal virgin olive oils from Extremadura and the relationship with oxidative stability. *IJMS* **2016**, *17*, 1960, doi:10.3390/ijms17111960.
15. Ramos-Escudero, F.; Morales, M.T.; Asuero, A.G. Characterization of bioactive compounds from monovarietal virgin olive oils: relationship between phenolic compounds-antioxidant capacities. *International Journal of Food Properties* **2015**, *18*, 348–358, doi:10.1080/10942912.2013.809542.
16. Becerra-Herrera, M.; Vélez-Martín, A.; Ramos-Merchante, A.; Richter, P.; Beltrán, R.; Sayago, A. Characterization and evaluation of phenolic profiles and color as potential discriminating features among spanish extra virgin olive oils with protected designation of origin. *Food Chemistry* **2018**, *241*, 328–337, doi:10.1016/j.foodchem.2017.08.106.
17. Quintero-Flórez, A.; Pereira-Caro, G.; Sánchez-Quezada, C.; Moreno-Rojas, J.M.; Gaforio, J.J.; Jimenez, A.; Beltrán, G. Effect of olive cultivar on bioaccessibility and antioxidant activity of phenolic fraction of virgin olive oil. *Eur J Nutr* **2018**, *57*, 1925–1946, doi:10.1007/s00394-017-1475-2.
18. López-Yerena, A.; Ninot, A.; Jiménez-Ruiz, N.; Lozano-Castellón, J.; Pérez, M.; Escribano-Ferrer, E.; Romero-Aroca, A.; Lamuela-Raventós, R.M.; Vallverdú-Queralt, A. Influence of the ripening stage and extraction conditions on the phenolic fingerprint of 'corbella' extra-virgin olive oil. *Antioxidants* **2021**, *10*, 877, doi:10.3390/antiox10060877.

19. López-Yerena, A.; Lozano-Castellón, J.; Olmo-Cunillera, A.; Tresserra-Rimbau, A.; Quifer-Rada, P.; Jiménez, B.; Pérez, M.; Vallverdú-Queralt, A. Effects of organic and conventional growing systems on the phenolic profile of extra-virgin olive oil. *Molecules* **2019**, *24*, 1986, doi:10.3390/molecules24101986.
20. Bayram, B.; Esatbeyoglu, T.; Schulze, N.; Ozcelik, B.; Frank, J.; Rimbach, G. Comprehensive analysis of polyphenols in 55 extra virgin olive oils by HPLC-ECD and their correlation with antioxidant activities. *Plant Foods Hum Nutr* **2012**, *67*, 326–336, doi:10.1007/s11130-012-0315-z.
21. Loubiri, A.; Taamalli, A.; Talhaoui, N.; Mohamed, S.N.; Carretero, A.S.; Zarrouk, M. Usefulness of phenolic profile in the classification of extra virgin olive oils from autochthonous and introduced cultivars in Tunisia. *Eur Food Res Technol* **2017**, *243*, 467–479, doi:10.1007/s00217-016-2760-7.
22. Gargouri, B.; Ammar, S.; Zribi, A.; Mansour, A.B.; Bouaziz, M. Effect of growing region on quality characteristics and phenolic compounds of chemlali extra-virgin olive oils. *Acta Physiol Plant* **2013**, *35*, 2801–2812, doi:10.1007/s11738-013-1312-z.
23. Ouni, Y.; Taamalli, A.; Gómez-Caravaca, A.M.; Segura-Carretero, A.; Fernández-Gutiérrez, A.; Zarrouk, M. Characterisation and quantification of phenolic compounds of extra-virgin olive oils according to their geographical origin by a rapid and resolutive LC–ESI-TOF MS method. *Food Chemistry* **2011**, *127*, 1263–1267, doi:10.1016/j.foodchem.2011.01.068.
24. Alkan, D.; Tokatli, F.; Ozen, B. Phenolic characterization and geographical classification of commercial extra virgin olive oils produced in Turkey. *J Am Oil Chem Soc* **2012**, *89*, 261–268, doi:10.1007/s11746-011-1917-6.
